# Locoregional Treatment for Early-Stage Breast Cancer: Current Status and Future Perspectives

**DOI:** 10.3390/curroncol30080545

**Published:** 2023-08-10

**Authors:** Sayeh Lavasani, Erin Healy, Kari Kansal

**Affiliations:** 1 Division of Hematology and Medical Oncology, UC Irvine, Orange, CA 92868, USA; 2 Department of Radiation Oncology, UC Irvine, Orange, CA 92868, USA; 3 Division of Breast Surgery, UC Irvine, Orange, CA 92868, USA

**Keywords:** breast cancer, locoregional treatment, de-escalation breast surgery

## Abstract

Background: The locoregional recurrence of breast cancer has been reduced due to the multidisciplinary approach of breast surgery, systemic therapy and radiation. Early detection and better surgical techniques contribute to an improvement in breast cancer outcomes. Purpose of Review: The purpose of this review is to have an overview and summary of the current evidence behind the current approaches to the locoregional treatment of breast cancer and to discuss its future direction. Summary: With improved surgical techniques and the use of a more effective neoadjuvant systemic therapy, including checkpoint inhibitors and dual HER2-directed therapies that lead to a higher frequency of pathologic complete responses and advances in adjuvant radiation therapy, breast cancer patients are experiencing better locoregional control and reduced local and systemic recurrence. De-escalation in surgery has not only improved the quality of life in the majority of breast cancer patients, but also maintained the low risk of recurrence. There are ongoing clinical trials to optimize radiation therapy in breast cancer. More modern radiation technologies are evolving to improve the patient outcome and reduce radiation toxicities.

## 1. Background

Due to the multidisciplinary approach to locoregional disease in breast cancer, the local recurrence and long-term outcome of breast cancer have improved. The decline in ipsilateral breast cancer recurrence is due to multiple factors including early detection, better surgical techniques, an effective neoadjuvant and adjuvant systemic therapy and adjuvant radiation. The improvement in local recurrence and long-term outcome has shifted the treatment toward an improvement in quality of life and decreased morbidities, such as lymphedema, which is achievable with de-escalation. There are ongoing clinical trials to de-escalate local therapy while omitting surgery and/or radiation in ductal carcinoma in situ and early-stage hormone-positive breast cancer in older adults [1,2]. In this review, we will evaluate the current approach to the locoregional treatment of breast cancer including surgical, systemic therapy and radiation treatment. We will also discuss the future directions.

## 2. Role of Surgery in Locoregional Control

### 2.1. Modern Breast Surgery

To understand where we are with treatment, we must review the past. The surgical treatment of breast cancer has evolved drastically since the Halsted radical mastectomy was established as the operation for breast cancer for most of the 20th century. The radical mastectomy included the breast, pectoralis major muscle and a level 1–3 axillary lymphadenectomy. Patients and surgeons began to voice dissatisfaction for this procedure in the mid1960s [3] when we began to develop a deeper understanding of histology and systemic therapies. This evolution came from a landmark clinical trial, NSABP B-04 Table 1 [4], comparing radical vs. total mastectomy followed by postmastectomy radiation therapy (PMRT) in node negative patients, or total mastectomy plus axillary lymph node dissection (ALND) in patients who converted to node positive. A 25-year follow-up showed that less surgery was equivalent in regard to disease-free survival (DFS), relapse-free survival (RFS), distant disease-free survival (DDFS) or overall survival (OS). The authors of this paper state “Although differences of a few percentages points cannot be excluded, the findings fail to show a significant survival advantage from removing occult positive nodes at the time of initial surgery or from radiation therapy”. This has now been shown repeatedly through multiple clinical trials that will be discussed.

### 2.2. Breast Conservation

The evolution of a radical mastectomy to less surgery was the first step toward improvements in the morbidity from lymphedema, pain, decreased range of motion and function. With the knowledge gained in the NSABP B-04 trial (Table 1)and women’s liberation movement, breast conservation became the focus in clinical trials. In addition, screening mammography was recommended by the American Cancer Society in 1976 in the United States for which breast cancers were found at an earlier stage and breast conservation became popular. NSABP B-06 (Table 1) compared a partial mastectomy with irradiation, a total mastectomy and a partial mastectomy alone. The patients that were enrolled, including those with and without axillary disease and tumors ≤ 4 cm, were randomly assigned to the three cohorts. Once again, at a 12-year follow-up, there was no difference in OS or DFS among the three cohorts. At a 10-year follow-up, 90% of the patients who underwent a partial mastectomy followed by radiation remained cancer free in the ipsilateral breast vs. 35% in the partial mastectomy alone group [4]. Breast conservation therapy has now become the dominant surgical treatment for patients with early-stage breast cancer. 

### 2.3. Acceptable Margin at Time of Resection

There has been much debate on the adequate margin needed at the time of surgical resection for breast cancer. This has ranged greatly in the literature from no ink on tumor to margins > 1 cm. There has also been debate between invasive and non-invasive breast cancer. In 2014, a consensus statement was issued after a systematic review and meta-analysis across all subspecialties including surgical oncologists, medical oncologists, radiation oncologists and breast pathologists. The consensus is that no ink on tumor minimizes the risk of invasive breast cancer recurrence and a 2 mm margin is required for pure ductal carcinoma in situ (DCIS) [9,10]. The American Society of Breast Surgeon’s official statement now follows these recommendations and is widely accepted as the standard of care.

### 2.4. Sentinel Lymph Node Biopsy and Avoidance of Completion Axillary Lymph Node Dissection

Through the twentieth century, we moved further from the radical mastectomy to partial mastectomy for early-stage breast cancer. Women were able to have outpatient surgery with minimal morbidity, although the standard was to perform a level 1 and 2 ALND for patients with invasive breast cancer. Moving from an ALND to a sentinel lymph node (SLN) biopsy to surgically stage the axilla in clinically node negative patients was another major step in the de-escalation of breast cancer surgical treatment. SLN biopsy was shown repeatedly to be safe in patients who had a negative SLN [11,12,13]. Before the ACOSOG Z0011 trial (Table 1), the recommendation was to proceed with a SLN biopsy in clinically node negative patients and if the lymph node was found to be histologically positive, the patient would undergo a completion ALND [14]. This trial examined the outcomes of patients who would undergo a SLN dissection without a completion ALND in the setting of positive SLN. Partial mastectomy margins were required to be negative after resection. Patients that were identified to have positive metastatic disease through H&E were randomized to no completion ALND or no further surgery. Patients who were noted to have matted lymph nodes, gross extranodal disease or ≥3 positive lymph nodes were excluded. At a median follow up of 6.3 years, there was no difference between the two groups in regard to locoregional recurrences and OS [6]. At the 9.25-year median follow-up, the same results were found [7]. These results are very similar to those of NSABP B-04 (Table 1) even though the patient populations are likely very different as those in B04 were all palpable at presentation. From these trials, and many more, we understand that not all axillary disease will progress to be clinically significant.

The results of the ACOSOG Z0011 trial have been widely adopted for patients undergoing a partial mastectomy with a clinically negative axilla and are considered a standard of care in the NCCN guidelines version 4.2023 for invasive breast cancer. Patients have greatly benefited from this shift in treatment. In a retrospective chart review conducted at the Memorial Sloan Kettering Cancer Center, they looked at how many axillary lymph node dissections were avoided based on the Z0011 criteria. Out of 793 patients, 84% underwent SLND alone while the remainder underwent ALND based on ≥3 SLN-positive, extra capsular extension and/or surgeon choice [15]. Upon further analysis, they found a predictor for ALND, which was an extranodal extension >2 mm, and not age, histology, lymphovascular invasion, receptor status nor radiation fields.

The question of the surgical treatment of the axilla in patients who are undergoing a mastectomy is still ongoing. Patients included in the Z0011 trial all underwent breast conservation with adjuvant breast irradiation. The tangential fields of radiation treated level 1 of the axilla and part of level 2. Due to this, there was concern that those patients undergoing a mastectomy who may not qualify for post mastectomy radiation therapy would have their axilla undertreated if any disease was noted in their pathology. The AMAROS trial (Table 1) [8] compared radiotherapy or surgery of the axillary after a positive sentinel lymph node in breast cancer. This multi-institutional, prospective, randomized European trial utilized the same inclusion and exclusion criteria as Z0011 but also had a cohort of 248/4806 patients who underwent a mastectomy. There were no significant differences between the treatment groups in DFS and OS in either the breast conservation or mastectomy group. We are awaiting the results of the A011202 clinical trial (Table 2) that is looking at ALND vs. axillary radiation for SLN-positive patients. 

There has been a conundrum for patients who undergo a mastectomy and are found to have axillary metastasis through H&E in their SLN. A multidisciplinary conversation is necessary to discuss the patient’s clinical scenario and the necessity of completion ALND versus axillary radiation versus both ALND and radiation therapy. 

### 2.5. Prevention of Lymphedema

There remain clinical scenarios where an ALND remains the standard of care. These include patients who present with a clinically positive axilla and do not undergo neoadjuvant systemic therapy, those with a failed SLN mapping, those with sentinel or axillary lymph nodes that remain positive after neoadjuvant chemotherapy, those with an axillary recurrence following previous breast cancer treatment, those with matted lymph nodes or gross disease at the time of SLNB, those with occult breast cancer only presenting with axillary metastasis, SLN-positive patients who fall out of the Z0011 criteria and those with inflammatory breast cancer (ASBrS consensus statement). Lymphedema is a concern for all patients undergoing a lymphadenectomy. This known morbidity has been reported through all clinical trials including this treatment of the axilla. In the AMAROS trial, although there was no difference in survival, there was a significant difference in morbidity for the patient in terms of lymphedema with the axillary radiotherapy having 11% lymphedema noted at 5 years compared to 23% in the ALND group. Singhal et al. reported on the use of a prophylactic lymphovenous bypass at the time of ALND for breast cancer. This showed a decrease in lymphedema from at least 20% to 3.1% in the patient population treated [16]. Further studies confirm the decrease in lymphedema to similar levels [17].

## 3. Neoadjuvant Systemic Therapy

The evolution of breast surgery has not occurred in a silo. The majority of the credit has to be given to the quickly changing and improving systemic therapy that we have available to our patients. From the trials discussed above one can see that more surgery does not equate to a better OS or DFS. Improved systemic therapy is what has improved survival in the treatment of breast cancer. As we better understand the biology of individual breast cancers and can personalize treatment, pathologic complete responses (pCR) have become more common especially with utilizing dual HER2-directed therapy in HER2-positive breast cancer patients and the use of pembrolizumab, an immune checkpoint inhibitor in triple-negative breast cancer patients in a neoadjuvant setting [18,19]. The neoadjuvant treatment of triple-negative breast cancer has escalated with the adding of pembrolizumab to taxane-based chemotherapy and carboplatin (TCb) followed by doxorubicin plus cyclophosphamide (AC) to improve pCR. In HER2-positive breast cancer patients, the HER2-directed therapy in a neoadjuvant setting has been escalated by adding pertuzumab to trastuzumab, but there is some evidence that we can de-escalate the chemotherapy portion of the neoadjuvant therapy and it may be possible to omit carboplatin +/− taxane chemotherapy from the combination of docetaxel, carboplatin, trastuzumab and pertuzumab (TCHP) that is currently the standard neoadjuvant regimen and to maintain the high pCR rate [20,21]. Some patients who undergo neoadjuvant systemic therapy are able to downstage their breast surgery from a mastectomy to a partial mastectomy based on their radiological and clinical response to the systemic therapy. Although some patients continue to opt for a total mastectomy, many have the option now of breast conservation [22].

This cohort of patients was excluded from trials such as AMAROS and Z0011 as there was concern over the false negative rate of sentinel lymph node biopsy after neoadjuvant chemotherapy. The ACOSOG Z1071 trial set to evaluate the false negative rate of sentinel lymph node biopsy after neoadjuvant chemotherapy [23,24]. This multi-institutional prospective trial enrolled 649 patients with T0-4 N1-2 M0 disease who underwent neoadjuvant chemotherapy. All of the patients underwent sentinel lymph node biopsy followed by a completion axillary lymph node dissection. Dual tracing agents were encouraged, and 525 patients had ≥2 SLN identified and removed. The false negative rate was found to be 12.6% which is larger than the accepted 10%. Although, when the removal of the clipped lymph node was also performed (107 patients) the false negative rate dropped to 6.8%, and the clipped lymph node was found to reflect the overall nodal status in 93% of the patients. 

ACOSOG 71071 and multiple other trials (SENTINA, SN FNAC) have shown similar results. Patients who are converted to a clinically negative axilla with neoadjuvant systemic therapy can undergo a Targeted Lymph node Dissection (TLND) at the time of surgery. If there is any residual cancer noted in the lymph nodes that are removed, a completion ALND is recommended. Using systemic therapy to de-escalate axillary surgery will save many patients the morbidity of an ALND. The alliance A11202 study compares patients who undergo neoadjuvant chemotherapy and have a positive SLN. Patients are randomized to either ALND with chest wall and local lymph node radiation or to radiation alone regardless of surgery. Johnson et al. recently reported the results of a feasibility study by the Exceptional Responders Clinical Trials Group to eliminate surgery. Thirteen patients with triple-negative or HER2-positive breast cancer who received neoadjuvant systemic treatment underwent a vacuum-assisted core biopsy post systemic therapy. Those with pCR (6 patients, 46.2%) did not have any surgery, but they received whole breast radiation therapy. The median follow-up of 44.3 months did not reveal any ipsilateral breast tumor recurrence. The expansion phase of this study is ongoing [25,26].

### 3.1. Omission of Sentinel Lymph Node Biopsy

The de-escalation of surgery in the axilla has been very important in reducing the morbidity of surgery. SLN biopsy is performed in all patients who present with a clinically negative axilla. In 2013, Hughes et al. published their results of the CALGB 9343 trial that looked at lumpectomy plus tamoxifen with or without irradiation in women aged 70 or older with early-stage breast cancer [27]. They found the addition of radiation therapy did not provide benefits in terms of OS, DFS or ultimate breast preservation. In a sub-analysis, the women who did not undergo surgical axillary staging, nor radiation therapy, experienced a 3% recurrence in the axilla. In 2016, the Society of Surgical Oncology released a Choosing Wisely guideline recommending sentinel lymph node omission in this patient population. The impact of this was examined in a retrospective review showing sentinel lymph node biopsy was not associated with recurrence (2.9% vs. 5.5%, *p* = 0.279), but radiation therapy did reduce recurrence (1.1% vs. 6.1%, *p* = 0.002) [28]. The omission of surgical axillary staging should be considered in this low-risk group and discussed in a multidisciplinary fashion to personalize the surgical approach for all patients.

### 3.2. Omission of Surgery after Neoadjuvant Chemothreapy

Although surgical techniques have changed drastically, there continues to be some morbidity that is unavoidable after surgery. Omitting surgery all together in some settings would be beneficial to patients if oncologically safe. Multiple retrospective trials [29,30,31,32,33] have been published comparing radiotherapy alone instead of standard breast conservation therapy after neoadjuvant chemotherapy. These studies were based on a clinical complete response (no palpable disease) and showed no significant difference in overall survival, but an increase in local regional recurrence in the radiation arm. Clouth et al. [34] was the first to introduce imaging to assess for radiologic complete response. They followed this with multiple core needle biopsies to evaluate for pathologic complete response. A total of 16 out of the 101 patients had a pCR and omitted surgery. There was no significant difference between disease-free survival or overall survival. There was a trend toward increased local regional recurrence in the radiation-only arm (12.5% vs. 9.5%) at 33.5 months.

More recently, multiple prospective studies have utilized an image-guided core needle biopsy to evaluate for pCR using ultrasound and mammography [35,36,37]. The false negative rate (FNR) was greater than 10% in most studies, but when using a thicker needle, limited tumor bed, MRI guided biopsy [38,39,40] and increasing the sample size taken, the FNR dropped to an acceptable level. Pfob et al. [41,42] shows that using a machine learning algorithm can help to predict pCR. The NOSTRA-Feasibility Study (NCT04118192) is looking at US-guided tumor core biopsy in women with ER negative, HER2-positive early-stage disease who undergo neoadjuvant chemotherapy plus dual-targeted anti-Her2 treatment to observe the FNR. This trial is estimated to be completed in 2025. Ongoing research is needed to know which patients may be able to omit surgery.

## 4. Role of Adjuvant Systemic Therapy in Locoregional Control

Multiple studies have shown the benefit of systemic therapy in reducing the locoregional recurrence (LRR) of breast cancer. NSABP B-24 was one of the first studies that was conducted and that showed a reduction in the rate of LRR with tamoxifen in patients with DCIS. Patients with DCIS were randomized to receive tamoxifen in an adjuvant setting after surgical resection and radiation therapy. At a 7-year follow-up, patients who received tamoxifen showed a 39% relative risk reduction of ipsilateral and contralateral breast cancer; both stage 0 and invasive cancer were reduced from 16% in the placebo group to 10% in the tamoxifen group (*p* < 0.0003). Both hormone-receptor-positive and negative DCIS patients were enrolled in this study. There was no benefit from adding tamoxifen for hormone receptor-negative patients. At a 10-year follow-up, patients maintained low LRR [43,44].

A combined analysis of 5 NSABP studies (B-13, B-14, B-19, B-20, B-23) that randomized early-stage, node-negative breast cancer patients to lumpectomy and whole breast irradiation with or without systemic therapy showed lower LRR in patients who received systemic therapy. Of the patients who did not receive any systemic therapy, 12.3% had a 12-year incidence of ipsilateral recurrence compared to patients assigned to tamoxifen alone, chemotherapy alone, and tamoxifen plus chemotherapy of which 6.7%, 6.4%, and 6.8% had ipsilateral recurrences, respectively [45,46]. 

The secondary analysis of the TAILORx randomized clinical trial that was reported by Sparano et al. showed that patients with a high Oncotype DX recurrence score of 26 to 100 who received adjuvant chemotherapy did extremely well and 91% had a 5-year freedom of breast cancer distant or locoregional recurrence [47].

## 5. Role of Radiation in Locoregional Control

Adjuvant radiation has been an integral component of breast conservation for several decades. In 2011, the Early Breast Cancer Trialist’ Collaborative Group (EBCTCG) meta-analysis showed that for node-negative patients, radiation reduced the risk of local recurrence from 31.0% to 15.6% at 10-years and reduced the 15-year risk of breast cancer death from 20.5% to 17.2% [48]. For node-positive patients, the benefit of radiation was even more dramatic. In these patients, radiation reduced the 1-year risk of recurrence by 5 times from 26.0% to 5.1% [48]. The trials included in this practice changing meta-analysis, however, were all conducted between the years 1976 and 1999. Since this time, many advancements in systemic therapy, surgery and even radiation for breast cancer have been made. These advancements compel us to re-evaluate the role of our respective fields in the optimization of cancer control and the minimization of treatment-related toxicity. 

### 5.1. Regional Nodal Irradiation

Regional nodal irradiation (RNI) is the radiation of the draining lymph nodes of the breast, which often includes the axilla (levels I–III), supraclavicular lymph nodes (SCL) and internal mammary lymph nodes (IMN), after either a mastectomy or breast conserving surgery. In the late 1990s, multiple trials established RNI after a mastectomy as the standard of care for women with node-positive or high-risk breast cancer [49,50,51]. In the early 2000s, the NCIC Clinical Trials Group (CCTG) MA.20 trial showed that RNI after BCS for women with either node-positive or high-risk node-negative breast cancer decreased locoregional recurrence and improved disease-free survival but showed no difference in breast-cancer mortality [52]. The treatment of the regional lymph nodes increases the complexity of adjuvant radiation planning and is associated with increased toxicity including skin toxicity, esophagitis, pneumonitis, lymphedema, cardiac toxicity, upper extremity dysfunction and thyroid dysfunction when compared to whole breast or partial breast radiation.

The treatment of the regional nodes in women with 1–3 positive lymph nodes had been a source of controversy for several years. In 2011, the EBCTCG sought to end the debate with a meta-analysis that showed that the radiation after BCS reduced any first recurrence from 58.2% to 38.3%, for an absolute 10-year gain of 19.9% [48]. In 2014, a second EBCTCG meta-analysis showed the benefit of RNI after a mastectomy in patients with 1–3 positive nodes [53]. The addition of radiation after a mastectomy resulted in a reduction in any first recurrence (34.2% vs. 45.7%), locoregional recurrence (3.8% vs. 20.3%), breast cancer mortality (42.3% vs. 50.2%) and death (53.5% vs. 56.5%). Considering these meta-analyses, RNI is considered the standard of care for patients with node-positive breast cancer following both BCS and a mastectomy. The increasing use of neoadjuvant systemic therapies and biomarkers in the treatment of breast cancer, however, is re-opening the debate regarding the role of radiation and its targets.

### 5.2. Target Volume Design after Neoadjuvant Chemotherapy

Neoadjuvant chemotherapy (NAC) is increasingly being used in the treatment of breast cancer. NAC can not only downstage tumors, making them more amenable to breast-conserving surgery (BCS), but also a tumor’s response to NAC can give insight into the tumor biology and prognosis [54,55]. It is not clear, however, whether NAC changes the risk of recurrence in the chest wall or regional lymph nodes. 

Two large, randomized trials are currently investigating the role of radiation therapy after NAC in patients with a clinically node-positive disease. The NSABP B-51/RTOG 1304 trial (Table 2) is evaluating the role of RNI in patients who are clinically node-positive prior to NAC, but who achieve a pathologic complete response (pCR) in the lymph nodes at the time of surgery (ypN0). The primary endpoint for this trial is a 10-year disease free survival. The accrual for this trial is currently closed and the results are expected in the near future. In contrast, the ALLIANCE-A011202 trial compares axillary lymph node dissection (ALND) to axillary radiation (ART) for patients with node-positive disease (ypN1) after NAC and sentinel lymph node biopsy (SLNB). The primary endpoint of this trial is a 5-year DFS, and it is expected to complete its accrual in 2024.

### 5.3. Impact of Biomarkers on Adjuvant Radiation

Following the practice changing TAILORX trial, molecular assays are increasingly being used to guide and individualize breast cancer treatment [56]. Patients with biomarker-low-risk breast cancer (hormone receptor-positive, HER2-negative and low-grade) have a low risk of distant recurrence [48]. Although the biomarker assays are intended to predict the risk of distant recurrence, it is unclear if they can be used to extrapolate the risk of local recurrence. 

Two randomized trials have been initiated that aim to use genetic biomarkers to guide the adjuvant radiation. NRG-BR007 (Table 2), also known as the “DEBRA” trial, is evaluating the omission of breast radiation following BCS in patients with stage 1, hormone receptor-positive, HER2-negative, breast cancer in patients with Oncotype recurrence scores (RS) ≤18. Similarly, the CCTG MA.39 (Table 2), also known as “TAILOR RT”, trial is a randomized trial of omission of RNI in biomarker low-risk node-positive breast cancer. For early-stage HER2-positive breast cancers who achieve a pCR to NAC, the NRG-BR008 (“HERO”) trial (Table 2) is a phase III randomized trial evaluating the omission of adjuvant radiotherapy. These trials are all currently accruing. Novel genomic signatures, such as the Profile for Omission of Local Adjuvant Radiation (POLAR), are currently being validated, but have yet to be incorporated into any major radiation trials [57].

### 5.4. Non-Standard Radiation Fractionations for Breast Cancer

Conventional whole breast radiation therapy is delivered in 25 fractions over 5 weeks. However, there is a growing interest in the use of non-standard radiation fractionations, such as hypofractionated whole breast radiation (HBRT) and ultra-hypofractionated whole breast radiation (UHBRT) to make radiation treatment more convenient for patients. The FAST-FORWARD trial compared HBRT (1 week) to conventional whole breast radiation (3 weeks) in patients with early-stage breast cancer. The trial found that HBRT was non-inferior to conventional whole breast radiation in terms of local control and toxicity [58]. The NRG RTOG 1005 trial compared HBRT with a concurrent boost to conventional whole breast radiation plus a sequential boost in patients with high risk early-stage breast cancer and it reported similar rates of local recurrence and a similar impact on physician-rated cosmesis. The ALLIANCE 221505 (RT CHARM) trial (Table 2) is a phase III randomized trial of hypofractionated post-mastectomy radiation with breast reconstruction.

### 5.5. Minimizing Treatment-Related Toxicity

Radiation oncology is also moving towards more individualized treatments that maximize the cancer outcomes and minimize the treatment-related toxicity. Modern radiation techniques such as intensity-modulated radiation therapy (IMRT), prone positioning and proton therapy have reduced the dose to normal tissues such as the heart and lung, thereby minimizing any potential radiation-related cardiac or pulmonary toxicities. Smaller target volumes seen in partial breast irradiation further minimize the dose to normal tissues for lower-risk patients. Through appropriately de-escalating RNI in those patients who do not derive benefit, by omitting radiation in select lower-risk patients, modifying radiation targets to account for smaller surgeries and utilizing modern radiation treatment planning, radiation oncology is adapting and evolving to improve breast cancer treatment for all patients. In the future, more modern radiation technologies, such as FLASH radiotherapy, may be able to spare normal tissue while maintaining the anti-tumor effectiveness. 

## 6. Discussion

Due to an improvement in surgical techniques, a neoadjuvant systemic therapy that includes checkpoint inhibitors in triple-negative breast cancer patients and dual HER2-directed therapy in HER2-positive breast cancer patients leads to an improved pCR plus advances in radiation therapy which have changed the outcome of breast cancer patients. With the increased use of neoadjuvant systemic therapy, breast cancer patients are having less aggressive surgeries. The management of axilla has changed tremendously. We can downstage the axilla and reduce the number of axillary node dissections even if the patient had clinically positive lymph nodes before starting the neoadjuvant chemotherapy, which turned into negative axilla post neoadjuvant chemotherapy. 

We are escalating neoadjuvant therapy by adding a checkpoint inhibitor (pembrolizumab) and dual HER2-directed therapy to neoadjuvant chemotherapy in triple-negative and HER2-positive breast cancer patients even though some studies have shown a de-escalation from the chemo portion of neoadjuvant systemic therapy in selected HER2-positive patients can maintain the high pCR rate, which is translated into an improvement in DFS and OS and a decrease in treatment-associated toxicities [59]. 

Breast cancer patients are experiencing less locoregional and systemic recurrences and an improvement in DFS and OS. Early-stage breast cancer patients with proper surgery, adjuvant systemic therapy and radiation have a 0.5% risk of local recurrence per year, which previously was reported as 1% [60]. The use of biomarkers in the radiation therapy of breast cancer patients is under study. More modern radiation technologies are evolving to improve the outcome of breast cancer patients and to reduce radiation-associated toxicities.

## 7. Conclusions

There will always be a role for breast surgery in the treatment of breast cancer, but we have shown repeatedly that less surgery is equivalent in regard to disease free and disease specific overall survival. The de-escalation of surgery has been made possible with the improvement of systemic therapy in a neoadjuvant setting. Adjuvant systemic therapy also plays an important role in reducing both the systemic and local recurrence of breast cancer. There are a number of controversies surrounding the use of radiation therapy in breast cancer. These controversies are being investigated in a number of clinical trials. Multiple ongoing studies are investigating how to utilize and validate biomarkers in the adjuvant radiation therapy of breast cancer patients. The results of these trials will help to inform the optimal use of radiation therapy in breast cancer. More modern radiation technologies are evolving to improve the patient outcome and reduce the radiation toxicities.

### Future Directions

The evolution for the loco-regional treatment of breast cancer has de-escalated in a short amount of time as systemic therapy has improved. This allows us to give patients more options and personalize their care. A robotic-assisted nipple sparing mastectomy and sensation sparing mastectomy are two new additions to our surgical arsenal to improve patients’ quality of life. 

In addition to omitting surgery all together, further studies are continuing to look at percutaneous treatment including cryoablation and radiofrequency ablation. We predict that the de-escalation/omission of radiation therapy in low-risk breast cancer patients will be on the horizon.

## Figures and Tables

**Table 1:** Key surgical clinical trials.

Study	Published	Study Arms	Endpoints	Outcomes
NSABP B-06 Fisher et al. [4]	NEJM 1995	Lumpectomy followed by XRT vs. lumpectomy vs. mastectomy	DFS OS	No difference
NSABP B-04 Fisher et al. [5]	NEJM 2002	Mastectomy followed by radiation in node-negative patients vs. mastectomy + ALND	DFS RFS DDFR OS	No difference
ACOSOG Z0011 Giuliano et al. [6,7]	Ann of surg 2010 JAMA 2011	ALND vs. no ALND in SLN-positive patients	locoregional recurrences OS	No difference
AMAROS Donker et al. [8]	Lancet Oncology 2014	Axillary surgery vs. radiation in patients with positive SLN	DFS OS	Similar

ALND: axillary lymph node dissection, DFS: disease free survival, RFS: recurrence free survival, DDFR: distant disease-free survival, OS: overall survival.

**Table 2:** Ongoing radiation clinical trials.

Study	Phase	Therapy	Population
NSABP B-51/RTOG1304 NCT01872975	III	RNI vs. no RNI	Clinically node-positive who achieve pCR post NAC
Alliance A011202 NCT01901094	III	ALND vs. axillary radiation	NAC and positive SLNB
NRG BR007 (DEBRA) NCT04852887	III	De-escalation of radiation after BCS	Stage I, HR+, HER2−, Oncotype ≤ 18
CCTG MA.39 TAILOR RT NCT03488693	III	Regional radiotherapy in biomarker low-risk, node-positive breast cancer	ER+, HER2− 1–3LN+, Oncotype ≤ 18
the NRG-BR008 (“HERO”) NCT05705401	III	BCS > adjuvant HER2-directed therapy followed by radiation vs. no radiation	early-stage, low risk, HER2-positive breast cancer
ALLIANCE 221505 (RT CHARM) NCT03414970	III	hypofractionated post-mastectomy radiation with breast reconstruction to evaluate safety and radiation-related complications	Patients who had a mastectomy and reconstructive surgery

pCR: pathologic complete response, ALND: axillary lymph node dissection, NAC: neoadjuvant chemotherapy, SLNB: sentinel lymph node biopsy, BCS: breast conserving surgery, HR: hormone receptor, ER: estrogen receptor, LN: lymph node.
